# Dosing of Ertapenem in an Extreme Obesity: A Case Report of 250 kg Patient

**DOI:** 10.1155/2017/5310768

**Published:** 2017-10-08

**Authors:** Jana Lass, Kadri Tamme, Karin Kipper, Joel Starkopf

**Affiliations:** ^1^Tartu University Hospital Pharmacy, Tartu, Estonia; ^2^Department of Microbiology, University of Tartu, Tartu, Estonia; ^3^Clinic of Anaesthesiology and Intensive Care, Tartu University Hospital, Tartu, Estonia; ^4^Department of Anaesthesiology and Intensive Care, University of Tartu, Tartu, Estonia; ^5^Institute of Chemistry, University of Tartu, Tartu, Estonia; ^6^Analytical Services International, St George's University of London, London, UK

## Abstract

Limited available data for dosing in obesity of the medicines used in this case are discussed, with the emphasis on ertapenem. The case illustrates the difficulties in dosing medicines to morbidly overweight patients. The number of such patients is increasing but data on adequate doses of medicines are scarce. We demonstrate that ertapenem 1,5 g i.v. once daily provided adequate drug exposure for susceptible bacteria in a 250 kg patient with normal renal function. The case suggests the usefulness of therapeutic drug monitoring of antibiotics, especially in critically ill patients.

## 1. Introduction

Obesity may influence many aspects of the pharmacokinetics (PK) of medicines such as the volume of distribution (*V*d) which could be increased due to increased lean body mass and increased adipose tissue, binding to plasma proteins, metabolism, and elimination. eGFR and tubular secretion have been reported to increase in obesity [1]. All these changes could affect the pharmacokinetics of antibacterials, the correct administration of which is a cornerstone of sepsis therapy [2].

Ertapenem is a highly protein-bound (>90%) and mostly renally eliminated carbapenem antibiotic with low volume of distribution, indicated for the treatment of intra-abdominal infections, community acquired pneumonia, and skin and soft tissue infections caused by susceptible bacteria [3]. The standard dose is 1 g per day, but there are very limited published data that conventional dosing of ertapenem may result in suboptimal concentrations and consequently lead to a risk of potential treatment failure in obese patients [4].

Although anticipated alterations in the PK of ertapenem could possibly lead to changes in dosing requirements, increased dosing might be associated with adverse effects. Reduced dosing of ertapenem is recommended to patients with renal failure and cases of central nervous system toxicity (seizures, encephalopathy) have been described in this patient population [5, 6]. Monitoring of plasma levels of ertapenem does not belong to the standard laboratory assays available at ICUs, and thus the dosing of the drug in critically ill obese patients remains a matter of speculations and doubtful estimates. In the present report we describe ertapenem plasma concentrations in a patient of approx. 250 kg of weight.

## 2. Case Presentation

A 58-year-old morbidly obese man was admitted to the ICU of Tartu University Hospital, Estonia, with bilateral bacterial pneumonia (CRP 341 mg/L, procalcitonin 0,41 mcg/L), acute respiratory failure, and sepsis. Attempt to assess the patient's body weight with ICU bed equipped with weight scales resulted in alarm that 250 kg is exceeded, while no other equipment for precise measurements of that range of weight was available. Taking his height of 178 cm, his body mass index is >85 kg/m^2^.* Enterobacter aerogenes* (ESBL negative; Ampc-positive; MIC 0,064 mg/L) was cultivated from tracheal aspirate and treatment was started with ertapenem 1,5 g per day.

**Figure 1:**
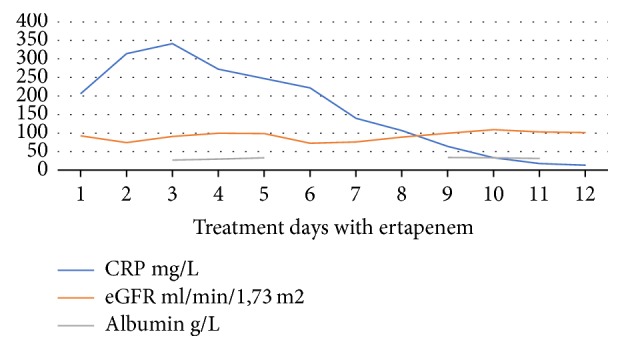
Values of C-reactive protein (CRP), estimated glomerular filtration (eGFR), and serum albumin during the treatment with ertapenem.

The dose of 1,5 g was chosen empirically. SPC recommends 1 g daily; we increased the dose arbitrarily with the assumption to somewhat increased volume distribution in this particular patient. The albumin levels and renal function were close to normal through the entire treatment period (Figure 1). Thanks to a research program we had the possibility of measuring ertapenem concentrations in the blood with validated ultrahigh performance liquid chromatography tandem mass spectrometry method [7]. The results became available with delay, and thus the data did not influence our treatment.

The measured maximum total concentration (*C*max) of ertapenem at steady state was 106.66 mg/L and through concentration (*C*min) 3.38 mg/L. According to the product information, the average *C*max and *C*min of ertapenem following a single 1 g intravenous dose in healthy young adults were 155 mg/L and 1 mg/L, respectively.

Pneumonia responded well to the treatment. After 10 days the inflammatory markers were markedly decreased (CRP 13 mg/L, procalcitonin 0,12 mcg/L) and clinical symptoms resolved.

## 3. Discussion

In this superobese patient, the maximum ertapenem serum concentration remained relatively low despite the administration of 150% of recommended daily dose. This may indicate that this subgroup of patients has somewhat higher volume of distribution (*V*_dss_) than subjects with normal body weight (*V*_dss_ in adults is approximately 8 litres (0.11 litres/kg)).

The literature data whether to use ideal, lean, or actual body weight for *V*_dss_ assessment are scarce. It has been shown that the total central compartment volume of ertapenem (*V*1) could be higher in morbidly overweight subjects (BMI, >40 kg/m^2^) compared to classes I-II obese subjects (mean increase in *V*1 of approximately 40% [4]). Recently, the population PK for ertapenem were assessed in the plasma of 6 female morbidly obese patients (BMI, 43.7 to 55.9 kg/m^2^). On day 2, the *V*dss was 57.8 litres in patients with a 53-kg fat-free mass [8]. Hence the effects of the obesity on the changes in *V*dss of ertapenem are not clear.

The higher *C*min of our patient might reflect lower rate of elimination, although the renal function according to eGFR was normal. Another possible explanation is the serum albumin effect, as ertapenem is highly protein-bound drug [9]. The serum albumin of the patient was at the lower end of the reference values, and thereby the free and measured fraction of the drug might have been slightly increased.

The PK parameter most correlated to the antibacterial efficacy of ertapenem is the time plasma concentration exceeding the MIC of the infecting organism [10]. For bactericidal effect, 35–40% of time above MIC has commonly been reported as sufficient for carbapenems [10, 11], although 50% was found necessary in critically ill patients [12]. The EUCAST MIC breakpoint for Enterobacteriaceae is ≤0.5 mg/L (susceptible). In our case the MIC value was very low (0,064 mg/L), and thereby the chosen dosing regimen resulted in 100% time over MIC (*f*_*T*>MIC_ = 100%).

Our results suggest that the 1,5 g dose of ertapenem could provide adequate drug exposure for superobese patients with normal renal function for achieving the pharmacodynamic targets. The case also demonstrates the usefulness of therapeutic drug monitoring of antibiotics, especially in critically ill patients. However, therapeutic drug monitoring of several antibiotics is not routinely available. There are several limitations of this study. There are many factors during the intensive care treatment and patient's clinical situation that could affect the generalizability of our results and to establish cause-effect relationship. Further research is needed on the adequate dosing of ertapenem in obese critical care patients.
